# Metaplasmidome-encoded functions of Siberian low-centered polygonal tundra soils

**DOI:** 10.1038/s41396-021-01003-y

**Published:** 2021-05-19

**Authors:** Adrian Gorecki, Stine Holm, Mikolaj Dziurzynski, Matthias Winkel, Sizhong Yang, Susanne Liebner, Dirk Wagner, Lukasz Dziewit, Fabian Horn

**Affiliations:** 1 Department of Environmental Microbiology and Biotechnology, Institute of Microbiology, Faculty of Biology, University of Warsaw, Warsaw, Poland; 2 GFZ German Research Centre for Geosciences, Section Geomicrobiology, Potsdam, Germany; 3 Institute of Biochemistry and Biology, University of Potsdam, Potsdam, Germany; 4 Institute of Geosciences, University of Potsdam, Potsdam, Germany; 5 Present Address: The German Federal Institute for Risk Assessment (BfR), Berlin, Germany

**Keywords:** Microbial ecology, Metagenomics, Soil microbiology, Next-generation sequencing

## Abstract

Plasmids have the potential to transfer genetic traits within bacterial communities and thereby serve as a crucial tool for the rapid adaptation of bacteria in response to changing environmental conditions. Our knowledge of the environmental pool of plasmids (the metaplasmidome) and encoded functions is still limited due to a lack of sufficient extraction methods and tools for identifying and assembling plasmids from metagenomic datasets. Here, we present the first insights into the functional potential of the metaplasmidome of permafrost-affected active-layer soil—an environment with a relatively low biomass and seasonal freeze–thaw cycles that is strongly affected by global warming. The obtained results were compared with plasmid-derived sequences extracted from polar metagenomes. Metaplasmidomes from the Siberian active layer were enriched via cultivation, which resulted in a longer contig length as compared with plasmids that had been directly retrieved from the metagenomes of polar environments. The predicted hosts of plasmids belonged to *Moraxellaceae*, *Pseudomonadaceae*, *Enterobacteriaceae*, *Pectobacteriaceae, Burkholderiaceae*, and *Firmicutes*. Analysis of their genetic content revealed the presence of stress-response genes, including antibiotic and metal resistance determinants, as well as genes encoding protectants against the cold.

## Introduction

Plasmids are mobile genetic elements and serve as examples of network-like structures of microbial adaptation [1]. Besides their basic structural genetic components that are crucial for maintenance, replication, and mobility, plasmids frequently carry an additional genetic load, with antibiotic and heavy-metal resistance being their most widely studied properties [2, 3]. Plasmids seem to be part of an ancient and successful strategy of ensuring the survival of a soil microbial population that is exposed to various environmental stresses [4, 5].

Plasmid-carried genes in environmental samples are potentially important in harsh climates as these environments are often endemic and highly vulnerable to the influence of external factors. The Arctic low-centered polygonal tundra soils represent extreme environments exposed to rapidly changing conditions due to seasonal droughts, frequent freeze–thaw cycling, excessive UV radiation, high or low pH, high osmotic pressure, and low nutrient availability [6–9]. The changing conditions are exacerbated by global warming. This makes polar regions interesting for investigating the potential role of horizontal gene transfer, which could be a major force behind bacterial diversification and adaptation to such challenging conditions.

Plasmids are composed of exchangeable genetic modules, which makes their classification difficult [10]. They may be classified based on their replication systems that enable an autonomous replication of plasmids within bacterial cells [11]. This sort of classification of replication initiation systems is called replicon typing and has been successfully applied in the past [11–13]. However, as many types of replication systems have yet to be identified, this classification system cannot be used for distinguishing plasmids of environmental isolates. Moreover, some plasmids might carry various replication systems, thus making it difficult to classify these complex replicons. Besides the known replication systems, the conjugal transfer modules, and well-characterized phenotypic genes (e.g., those that confer antibiotic and heavy-metal resistance), a large proportion of plasmid DNA encode currently unknown functions. One way to annotate these genes and reveal their putative/hypothetical functions is by assigning their products to protein families of clusters of orthologous groups (COGs). Each COG family consists of individual orthologous genes/proteins from at least three lineages that have the same function. This yields a number of functional predictions for poorly characterized genomes, thereby providing a framework for functional and evolutionary genomic analyses [14].

Sequencing plasmids and the complete genomes of isolated strains have provided insights into the plasmid-mediated plasticity of soil bacteria [15]. The lack of complex studies investigating the environmental pool of plasmids is due to difficulties in their retrieving from metagenomic data and analyzing the plasmids by applying primarily culture-dependent methods [5, 16–19]. These methods include plasmids captured from soil via conjugation, the selective amplification of circular DNA using phi29 DNA polymerase, and the isolation of plasmid DNA from cultivable bacterial isolates.

Plasmids obtained from cultivable bacteria originating from the polar regions have been found to carry cold shock proteins, UV radiation protection genes, and genes that code for protection against reactive oxygen species [20]. These accessory genes constitute a reservoir of diverse genetic determinants, whose presence may serve as an evolutionary advantage that is essential for appropriate adaptation to harsh environmental conditions [5]. For years, High-Arctic soils were conceived as pristine and uninfluenced by human activities, but they are now increasingly exposed to various pollutants from industry, agriculture, and other sources [21, 22]. The genes encoding antibiotic and metal resistance, which are frequently found within plasmids, are interesting indicators of this contamination. Importantly, antibiotic resistance genes have been detected in plasmids from various polar regions; however, not much is known about antibiotic resistance in plasmids from the High-Arctic soils [23–27].

In this study, we enriched environmental microbial communities from two Siberian low-centered polygonal tundra soil samples and investigated their metaplasmidomes. We compared the genetic repertoire with other Arctic and Antarctic plasmid-carried genes that have been directly retrieved from metagenomes in order to explore the diversity and biological functions of the metaplasmidomes.

## Material and methods

### Site description

Two sampling sites (S9: 72°37021 N 126°47942 E and S11: 72°36983 N 126°47791 E) in the polygonal tundra on Samoylov Island in the Lena Delta (Siberian Arctic) were investigated. The sampling took place in July 2016 as part of the Lena Delta expedition LENA2016 [28]. The two sites represent low-centered polygons with a water-saturated center, which is typical of Arctic polygonal tundra [29, 30]. The elemental composition was measured using CHNS Elemental Analyzer Flash EA1112 (Thermo Finnigan Italia SpA) in mg g^−1^ dry mass of soil. Both sites showed the highest amount of carbon and nitrogen in the upper 10 cm. Site S9 showed a carbon content of 402.2 (±121.5) mg g^−1^, nitrogen content of 47.5 (±16.6) mg g^−1^, hydrogen content of 81.8 (±33.3) mg g^−1^, and sulfur content of 55.5 (±16.6) mg g^−1^. For site S11, the carbon content was 98.6 (±17.2) mg g^−1^, and the nitrogen content was 1.1 (±0.2) mg g^−1^. The hydrogen and sulfur contents were below the detection limit for site S11.

The samples were collected from the upper 10 cm of the polygon center using sterile steel cores and then placed into sterile 50-mL tubes. The samples were shipped back to Germany under frozen conditions and were kept frozen until further processing. Site S9 was water-saturated except for the uppermost 2–4 cm and had an active-layer depth of 42 cm at the time of sampling. Site S11 was water-saturated except for the uppermost 5–6 cm and had an active-layer depth of 40 cm at the time of sampling.

In addition, three samples, namely OD2, OD3, and OD5, were taken from a polygon center on Samoylov Island in the Lena Delta (in the vicinity of S9 and S11 sites). These three samples represented active layer, transition layer, and permafrost, respectively. DNA extracted from these samples were subjected to shotgun metagenomic sequencing, and the results were combined with S9 and S11 metaplasmidome data.

### Enrichment for plasmid isolation

**Fig. 1:**
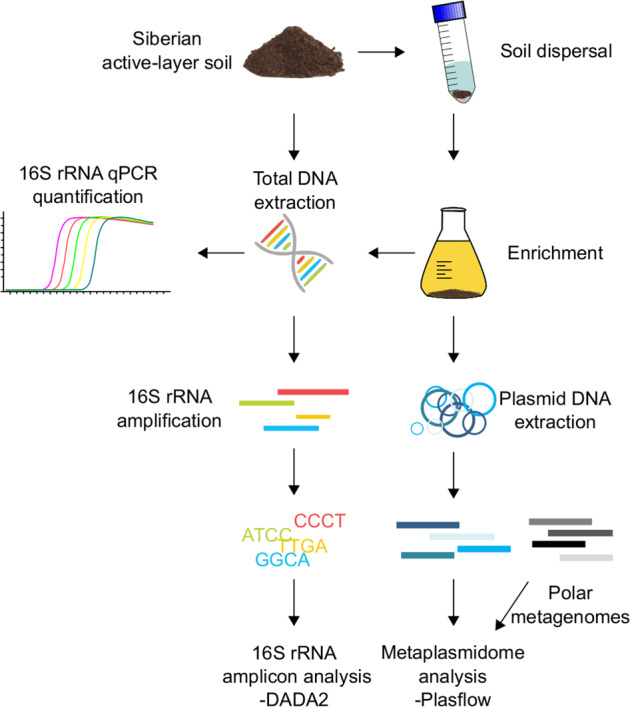
Depiction of the applied methodology for analyzing the soil metaplasmidome. Total DNA was extracted from the raw soil and enriched soil. The bacterial abundance was quantified by quantitative PCR (qPCR), and 16S rRNA genes were amplified and sequenced using the Illumina platform. The soil inoculum was dispersed via glass bead treatment. Plasmid DNA was extracted from the enrichment, and the metaplasmidome was sequenced.

We dispersed 5 g of soil in a 45-mL pyrophosphate buffer with sterile glass beads for 30 min on a rotor at 120 rpm and subsequently vortexed it for 2 min. The dispersed soil/buffer mixture was spun down, and 4 mL was inoculated in 200 mL of R2A medium, which equaled 2.0% inoculum [31]. The soil microbiota was enriched for 48 h on a shaker at 110 rpm. After 48 h of cultivation at 22 °C, plasmid DNA extraction was performed from the liquid medium using a Plasmid Midi Kit (Qiagen). The workflow is summarized in Fig. 1. It is important to note that, according to the manufacturer, plasmids up to approximately 150 kb can be purified using this kit, but replicons larger than 45–50 kb may exhibit reduced elution efficiencies. This might introduce a bias into the experiment; however, based on our experiences, this procedure turned out to be most efficient for plasmid isolation, compromising reasonable plasmid extraction rate and the purity of extracted DNA.

### Metaplasmidome and metagenome sequencing

Two metaplasmidome libraries were constructed using MiSeq Reagent Kit v3 (Illumina). The libraries were later sequenced in 2 × 300 bp paired-end mode using an MiSeq instrument (Illumina) by DNA Sequencing and Oligonucleotide Synthesis Laboratory IBB PAN (oligo.pl, Warsaw, Poland). The metagenome sequencing of OD2, OD3, and OD5 was performed on the HiSeq 2500 (Illumina) system at Eurofins Scientific (Germany).

### Total DNA extraction and amplicon sequencing

The total DNA was extracted from ~300 mg of frozen soil samples and from the enrichments using 5 mL of R2A enrichment cultures. The PowerSoil DNA isolation kit (MO BIO Laboratories, Inc.) was used to optimize the extraction from the untreated soil samples, and the Fast DNA SPIN Kit (MP Biomedicals) was used to extract the total DNA from the liquid phase enrichments. The DNA concentrations were determined using the Qubit 2.0 Fluorometer (Invitrogen).

The bacterial 16S rRNA genes were amplified from the soil, both without enrichment and after enrichment, using the primers Bac341F [32] and Bac806R [33]. Each reaction contained Optitaq polymerase (Roboklon GmbH) at a concentration of 1.25 U. A template concentration of 10 ng and a total of 25 µL of reaction volume were used. PCR conditions comprised an initial denaturation at 95 °C for 5 min, followed by 35 cycles of denaturation (95 °C for 30 s), annealing (56 °C for 30 s), elongation (72 °C for 1 min), and a final extension step of 72 °C for 10 min.

Libraries were demultiplexed using Cutadapt [34], in which primer sequences had a maximum error rate of 10.0% and barcodes were required to have a Phred score higher than Q25 without any mismatches. Samples were further processed using the DADA2 pipeline [35]. Read sequences were truncated (250/200 forward/reverse) and quality-filtered before generating the error model. After dereplication, sample inference, and merging of the paired-end reads, merged sequences were required to have a minimum length of 200 bp. The orientation of the sequences was standardized by calculating the Hamming distances of the sequences and their reverse complement. The sequence table was created, and chimeras were removed using a de novo approach. The amplicon sequence variants (ASVs) were assigned to the SILVA taxonomy database (v132) [36] using VSEARCH [37] as utilized in the framework of QIIME2 [38]. Raw sequencing data are available at the European Nucleotide Archive (ENA) under BioProject accession number PRJEB33528 and sample accession numbers ERS3581726–ERS3581733.

Absolute read counts were transformed to relative abundances to standardize the data and account for different sequencing depths. All ASVs not assigned to *Bacteria* on the highest taxonomic level were removed prior to statistical analysis. Variations in ASV composition between samples and their diversity were assessed using PAST 3.12 software [39].

### Quantification of bacterial 16S rRNA gene abundance

Quantitative PCR was performed using the CFX Connect Real-Time PCR Detection System (Bio-Rad Laboratories) as described previously [28]. Primers (100 µM) Eub341F [32] and Eub534R [33] were used to amplify bacterial 16S rRNA genes by applying HiFi SYBR Green reagent (KAPA Biosystems). qPCR reactions were run in triplicates. The specificity of qPCR reactions was verified by melt curve analysis. Genomic standards of *Escherichia coli* cultures were included in each qPCR run to ensure linearity and the expected slope values of the Ct/log curves. PCR efficiency—based on the standard curve—was calculated using the CFX manager software (Bio-Rad Laboratories) and varied between 95.0 and 100.0%. Bacterial 16S rRNA gene copy numbers were calculated per gram of dry weight of sediment (gdw^−1^).

### Mining of metaplasmidomes

The sequencing results of metaplasmidomes from our enrichment cultures (S9 and S11), three unpublished Siberian metagenomes from Samoylov Island (OD2, OD3, and OD5), and 20 previously published metagenome datasets retrieved from the ENA (12 Arctic and 8 Antarctic metagenomes), obtained by paired-end mode using the HiSeq platform (Illumina) with at least 1 000 000 reads, were processed and assembled using MEGAHIT with default values (Table 1) [40]. Contigs smaller than 1000 bp were excluded. The putative metaplasmidomes were extracted using the PlasFlow software with a threshold of 0.7 [41]. The coding regions from the metaplasmidomes were eventually obtained using the Prodigal software using the meta flag [42].

**Table 1:** Metadata for metagenomic datasets used in this study.

SRA accession number	Biome	Area	Geographical region	Number of contigs after the PlasFlow	Average size of contigs after the PlasFlow (bp)	Recovery rate [%]	Read counts
SRX3369487	Polar desert sand microbial communities	Antarctic	Dry Valley	102 427	2001 (1000–157 004)	4.58	119 487 077
SRX3369477	Polar desert sand microbial communities	Antarctic	Dry Valley	76 132	2197 (1000–153 153)	4.39	141 231 096
SRX3048371	Polar desert sand microbial communities	Antarctic	Dry Valley	108 306	2001 (1000–261 617)	4.29	102 383 866
SRX3369488	Polar desert sand microbial communities	Antarctic	Dry Valley	41 124	2028 (1000–87 705)	4.21	51 906 622
SRX3369435	Polar desert sand microbial communities	Antarctic	Dry Valley	59 104	1897 (1000–50 741)	4.09	62 189 424
SRX3369431	Polar desert sand microbial communities	Antarctic	Dry Valley	51 981	1865 (1000–41 997)	3.32	101 982 500
SRX3372244	Polar desert sand microbial communities	Antarctic	Dry Valley	567	1544 (1000–6495)	1.34	79 928 880
SRX3521479	Rhizosphere of *Colobanthus quitensis* growing with *Deschampsia antarctica*	Antarctic	Livingston Island	4112	1454 (1000–29 811)	0.42	3 715 503
SRX5358028	Peat permafrost microbial communities	Arctic	Abisko	375 218	2137 (1000–195 792)	4.95	353 315 087
ERX2451526	Marine sediment	Arctic	Bulunsky District	151 250	1661 (1000–41 191)	3.03	126 019 168
ERX2451524	Marine sediment	Arctic	Bulunsky District	163 197	1621 (1000–64 577)	2.84	136 114 901
ERX2451525	Marine sediment	Arctic	Bulunsky District	186 277	1680 (1000–50 886)	2.82	151 772 352
ERX2451528	Marine sediment	Arctic	Bulunsky District	53 181	1542 (1000–34 571)	2.78	47 357 589
SRX2163492	Microbial communities from late Pleistocene permafrost sediments	Arctic	Nizhnekolymsky District	206 262	1462 (1000–30 568)	2.42	79 379 572
SRX2910045	Arctic peat soil microbial communities	Arctic	Barrow	21 566	1751 (1000–50 583)	2.38	168 696 021
ERX2451527	Marine sediment	Arctic	Ust-Yansky District	42 167	1515 (1000–39 027)	2.02	54 640 484
SRX2163490	Microbial communities from late Pleistocene permafrost sediments	Arctic	Bykovsky Peninsula	99 212	1454 (1000–26 998)	1.88	58 879 198
SRX2163491	Microbial communities from late Pleistocene permafrost sediments	Arctic	Gydan Peninsula	23 432	1411 (1000–11 348)	1.32	65 193 260
SRX763249	Permafrost microbial metagenome	Arctic	Nizhnekolymsky District	4	2525 (1213–5505)	0.31	1 000 000
SRX751044	Permafrost microbial metagenome	Arctic	Nizhnekolymsky District	17	1410 (1023–5505)	0.25	1 000 000
S9^a^	Active-layer permafrost	Siberia	Samoylov Island	106	9745 (1000–140 607)	40.30	194 560
S11^a^	Active-layer permafrost	Siberia	Samoylov Island	16	9463 (1480–38 611)	34.78	152 846
OD2^b^	Active-layer permafrost	Siberia	Samoylov Island	25 512	1497 (1000–33 035)	1.24	71 268 645
OD3^b^	Transition layer	Siberia	Samoylov Island	15 811	1454 (1000–37 991)	0.80	84 950 816
OD5^b^	Permafrost layer	Siberia	Samoylov Island	15 845	1427 (1000–26 135)	0.86	70 839 591

Values in round brackets correspond to minimal and maximal length of contigs.

^a^Datasets (metaplasmidomes) obtained from the enrichment cultures.

^b^Siberian metagenomes from vicinity of S9 and S11 sites from Samoylov Island (unpublished).

The number of plasmid contigs, the size of the contigs, and the plasmid recovery rate (Eq. 1)—defined by the proportion of contigs classified as plasmids out of all obtained contigs after assembling—are included in Table 1. The recovery rate is a suitable indicator of the recovery of plasmid contigs exclusively from the whole metagenome sequencing and can be interpreted as an efficiency indicator for the applied plasmid enrichment in the proposed metaplasmidome-targeted sequencing.

1 $$	\,\;{\rm{Number}}\;{\rm{of}}\;{\rm{contigs}}\;{\rm{after}}\;{\rm{filtration}}\\ {\rm{Recovery}}\;{\rm{rate}} =	\, \frac{ {\rm{with}}\;{\rm{the}}\;{\rm{PlasFlow}}\;[32]\qquad\quad\qquad }{{\rm{Number}}\;{\rm{of}}\;{\rm{contigs}}\;{\rm{after}}\;{\rm{assemblig}}} \times 100\% \\ 	\, \;{\rm{raw}}\;{\rm{reads}}\;{\rm{with}}\;{\rm{MEGAHIT}}\;[38]$$

### Identification of potential plasmid hosts

Two methodological approaches were used for the identification of potential plasmids’ hosts based on plasmid contigs. Initially, a lowest common ancestor (LCA) approach was used to assign contigs to the host (BASTA software with defaults settings) [43]. In the second approach, sequences of predicted proteins encoded by plasmid-carried genes were used as a query for BLASTp [44] against the non-redundant NCBI database. Then, information about the putative host was extracted by supplementing best BLASTp hits output search with “ssciname” specifier enabling extracting information about the source organism. Retrieved information was curated and extended with missing taxonomic levels. Particular plasmid-contig was assigned to a specific host only if more than 75.0% of proteins encoded within that contig were assigned to one genus/taxon.

### Classification of cluster of orthologous groups

In order to perform a functional annotation of the obtained plasmid contigs, the classification to orthologous groups was performed [45]. The COGs were assigned to each gene by a local RPS-BLAST search against the database with an 1e–05 *e*-value threshold by considering only the best BLAST hits [44]. Classified genes were additionally checked for similarity using BLASTn [44] with the specific threshold (percent identity: 75.0%; query coverage: 50.0%) against 18 919 full plasmid sequences that had been retrieved from the NCBI database on September 18, 2019. This checking was performed in order to evaluate the prevalence of Siberian metaplasmidome gene clusters in reference to experimentally confirmed full plasmid sequences.

### Identification of replication initiation protein

Predicted replication initiation proteins were classified according to their sequence similarity to plasmid replication proteins retrieved from the NCBI database. Automatically extracted sequences were additionally subjected to manual annotation with BLASTp and BLASTn tools available at the NCBI website, using default settings [44]. A similarity network was constructed based on an undirected weighted topology using numeric values corresponding to the average identity and query coverage of nucleotide sequences of confirmed genes related to genes encoding plasmid replicases obtained after alignment of sequences using BLAST tool [44]. Genes are represented as nodes and cluster in the network according to their weight (higher average value of identity and query coverage). Visualization of the network has been prepared using the Gephi software [46] by a combination of Reingold–Fruchterman and ForceAtlas 2 layouts [47].

### Establishing a database of stress-response genes

A database of stress-response genes (StressDB) was created to facilitate annotation of plasmid genes and to compare the content of the stress-response genes from the metaplasmidomes with plasmid contigs retrieved from metagenomes of polar environments. The StressDB was constructed by the compilation of publicly available data from known databases (i.e., CARD [48] and BacMet [49]) and an ad hoc literature search (performed until March 15, 2019), targeting cold-response genes/proteins (the sequences of these genes/proteins were extracted from a publicly available database, i.e., NCBI or Uniprot, using their accession numbers). In the database, only genes with confirmed functionality in cold protection, resistance to antibiotics, and metal metabolism were included. The StressDB is available under the following address: http://ddlemb.com/bioinformatic-tools-and-databases/. The compiled database contains 2451 sequences of genes encoding stress-response genes, including (i) 2191 antibiotic-resistant genes, (ii) 119 heavy-metal resistant genes, (iii) 49 reactive oxygen species protection genes, (iv) 30 UV radiation protection genes, (v) 23 cold shock genes, (vi) 13 antifreeze genes, (vii) 13 osmoregulatory genes, (viii) 4 phasin genes, (ix) 3 genes encoding trehalose synthetases, (x) 3 genes encoding hydroxyalkanoic acid synthetases, and (xi) 3 genes encoding ice nucleation proteins.

Similarity searches against the database were performed via BLASTp [44] using specific thresholds (query coverage per high-scoring segment pair of at least 75.0% and a percent identity of 50.0%). Only the best BLASTp hits were taken for further analysis. Data transformation and visualization were performed using the Python Matplotlib 3.1.1. package [50].

## Results

### Metaplasmidomes from enrichment cultures versus plasmid contigs retrieved from metagenome datasets

For the comparison of the efficiency of plasmid recovery from enriched metaplasmidomes and shotgun metagenomes retrieved from the databases, 20 polar metagenome datasets were assembled and analyzed. It was shown that the average size of the contigs retrieved from the enriched Siberian metaplasmidomes (obtained in this study) was 9604 bp, which is more than five times the contig length of plasmids directly extracted from metagenomes of polar environments. The average size of contigs assigned as plasmids by PlasFlow [41] was similar for Arctic metagenomes (1874 bp) and Antarctic metagenomes (1681 bp). The number of plasmid contigs obtained from metagenomes of polar environments after the PlasFlow filtration varied significantly, ranging from 4 (SRX763249) to 375 218 (SRX5358028). The recovery rate was highest (5.0%) for plasmids from an Arctic peat soil metagenome (SRX5358028) and lowest (0.3%) for those from an Arctic permafrost metagenome (SRX751044). Since we extracted plasmid DNA before analyzing the metaplasmidomes from our enrichments, the recovery rates for enriched metaplasmidomes were much higher, i.e., 34.8% and 40.3% for S11 and S9, respectively (Table 1). In addition, we compared Siberian metaplasmidomes obtained by enrichment with those directly extracted from Samoylov Island metagenomes (i.e., three datasets—OD2, OD3, OD5—obtained from samples collected in the vicinity of S9 and S11 sites). Results of this analysis showed that the average size of contigs for these datasets equals 1457 (±37.6) bp, whereas the recovery rate ranged from 0.8 to 1.2% (Table 1).

### Taxonomic structure of initial bacterial community and proportion recovered from enrichment

**Fig. 2:**
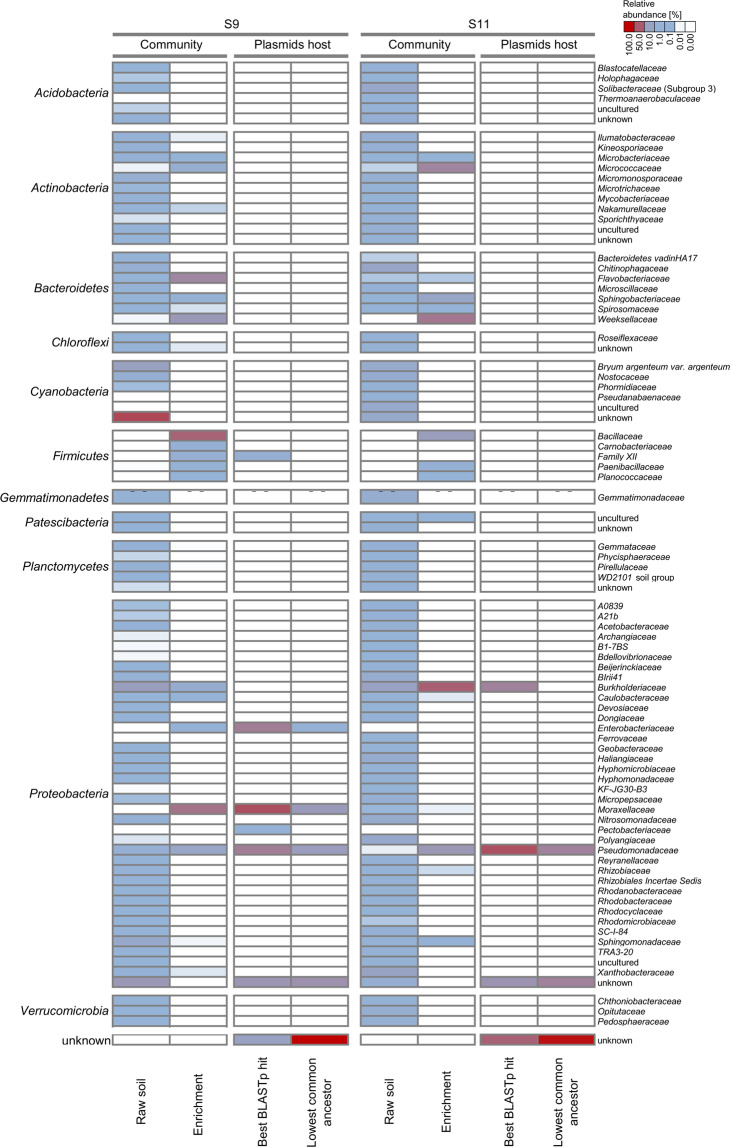
Heat map presenting taxonomic assignments to raw and enriched microbial communities as well as to potential plasmid hosts. The LCA and best BLAST hit approaches were applied. Cut-off: >0.2%.

From the original soil samples, 1506 and 1949 bacterial ASVs were identified in S9 and S11, respectively, with a bacterial 16S rRNA gene abundance of 1 × 10^7^–7.8 × 10^8^ bacterial gene copies gdw^−1^. The bacterial community of site S9 was dominated by *Cyanobacteria* (50.2%) and *Proteobacteria* (32.9%) of the family *Burkholderiaceae* (8.3%). The bacterial community of site S11 was equally dominated by *Proteobacteria* (45.0%) of the family *Burkholderiaceae* (6.6%) and *Xanthobacteraceae* (6.2%) (Fig. 2).

After enrichment, in S9- and S11-originated samples, 98 and 62 bacterial ASVs were identified, respectively, and a similar diversity between the two sites was observed, with *Proteobacteria* (S9: 34.2%; S11: 43.5%), *Bacteriodetes* (28.5% and 28.4%), *Firmicutes* (35.6% and 10.2%), and *Actinobacteria* (2.7% and 17.7%) dominating the bacterial enrichments. In site S9, *Proteobacteria* were dominated by representatives of the family *Moraxellaceae* (24.9%), *Pseudomonadaceae* (5.3%), and *Burkholderiaceae* (3.0%). *Bacteriodetes* were found to be dominated by the families *Flavobacteriaceae* (17.3%) and *Weeksellaceae* (10.2%). In site S11, *Proteobacteria* were dominated by the families *Burkholderiaceae* (32.2%) and *Pseudomonadaceae* (10.6%). Within the *Bacteriodetes*, the families *Weeksellaceae* (22.9%) and *Spingobacteriaceae* (5.4%) dominated. In both studied environments, *Actinobacteria* were mostly represented by *Micrococcaceae* (S9: 2.3%; S11: 17.7%). In both environments, a significant increase in the number of representatives of *Firmicutes* (and especially *Bacillaceae*) was observed after enrichment. In addition to changes in the microbial community, a significant drop in alpha diversity in the enrichments was shown by both the Shannon diversity index (S9: 4.8–3.5 and S11: 6.9–3.1) and the Chao1 factor (S9: 2531–717.3 and S11: 3042–176).

### Bacterial plasmid hosts

For the assignment of potential hosts for the plasmid contigs, two methodologies (LCA and best BLASTp hit) were applied. The LCA algorithm allowed classification of only 22.4% (for S9 metaplasmidome) and 15.4% (for S11) contigs to the genus level. It was shown that the contigs potentially originated from *Pseudomonas* spp. (for S9 and S11) and *Acinetobacter* sp. (for S9). By applying the LCA method, less than 68.0% (S9) and 63.0% (S11) of contigs could be classified, even at the phylum level. This indicated that the algorithm works poorly for the studied environments (Fig. 2).

By applying the best BLASTp hit methodology, it was shown that for the S9 metaplasmidome, the predicted hosts of plasmids are *Moraxellaceae* (37.7% of plasmid contigs), *Pseudomonadaceae* (20.8%), *Enterobacteriaceae* (20.8%), *Pectobacteriaceae* (0.9%), and *Firmicutes* (Family XII; 1.9%), while for S11, the potential hosts are *Pseudomonadaceae* (37.5%) and *Burkholderiaceae* (18.8%).

In both methodologies, the majority of plasmid contigs were not classified to any known taxa.

### Identification of replication initiation genes

To support the potential plasmid host identification carried out by the best BLASTp hit and the LCA method, we have identified the replication initiation genes within the plasmid contigs and assigned them using the best BLAST hit approach to the specific taxa. Within the enriched metaplasmidomes from Siberia, 41 and 7 replication genes were found for S9 and S11 metaplasmidomes, respectively. The majority of these genes (95.8%) were assigned to *Gammaproteobacteria* (mostly *Acinetobacter* and *Pseudomonas*). In the “reference” Arctic and Antarctic metagenomes retrieved from the ENA databases, the replication initiation genes were mostly assigned to *Alphaproteobacteria* (51.0%), *Gammaproteobacteria* (13.4%), and *Clostridia* (6.9%).

**Fig. 3:**
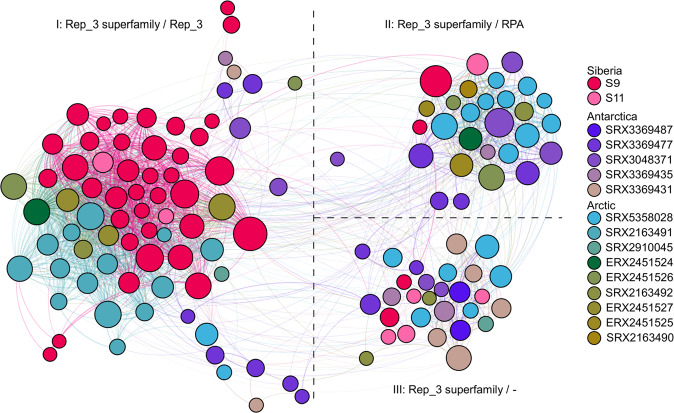
Protein-based similarity network of replication initiation proteins retrieved from metagenome datasets and enrichment-culture metaplasmidomes. Each node represents a single replication initiation protein (in total 133 proteins were analyzed), while edges correspond to the summarized quantity of reciprocally similar proteins. The colors denote the geography of the plasmids (red: Siberia; blue-green: Arctic; purple: Antarctica). The size of the node is proportional to the value of the betweenness centrality, which corresponds to how much a given node is in-between others, i.e., what is the number of the shortest paths that pass through the node when connecting two other nodes. In addition, nodes were color-shaded by their metagenome ID (Table 1).

A protein-based similarity network was constructed to compare the identified replication systems. Within the obtained network, each node represents a single replication initiation protein, and each edge corresponds to the homology between two proteins above a given threshold. According to the network, three main clusters were formed (Fig. 3). All three clusters belong to the Rep_3 Superfamily (cl19398). The first cluster contains RepB-like replication initiation proteins (pfam01051, COG5525) and consists of proteins putatively originating from *Gammaproteobacteria* (for Siberian metaplasmidomes) and *Clostridium* (for Arctic metagenomes). The second cluster consists of proteins of the RPA family (pfam10134, no specific COG). Within this cluster, mainly proteins that putatively originate from *Proteobacteria* were found. The third cluster gathers replicases putatively originating from various taxa, including *Acidobacteria*, *Actinobacteria*, *Chloroflexi*, *Cyanobacteria*, *Nitrospirae*, and *Proteobacteria*. Proteins in the third cluster could only be assigned to the Rep_3 superfamily (cl19398), with no specific hits to a particular group/family (Fig. 3).

### Analysis of orthologous gene families

**Fig. 4:**
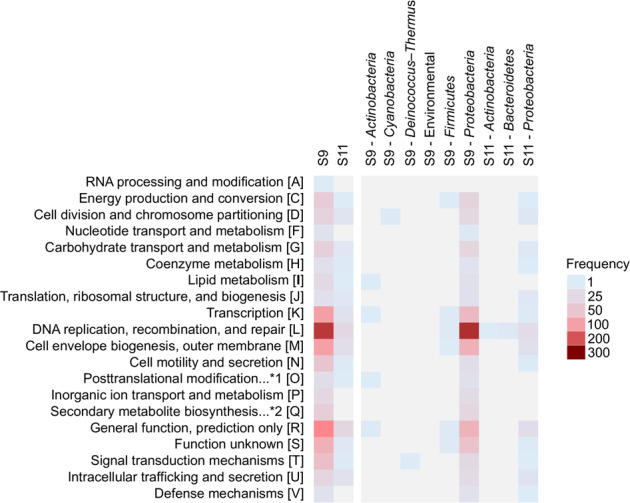
The number of genes from site S9 and S11 classified by COG functional categories. Letters in square brackets refer to abbreviations for the COG functional categories. *1—posttranslational modification, protein turnover, chaperones, *2—secondary metabolites biosynthesis, transport, and catabolism.

Genes predicted within S9 and S11 metaplasmidomes (Fig. 4) were blasted against the COG database [45] using RPS-BLAST with an *e*-value threshold of 1e–05. A total of 984 for S9 and 129 for S11 were assigned to the 1107 and 139 COG categories (multiple assignments of some genes occurred), respectively. In addition, classified coding sequences were checked for similarity with BLASTn [44] using a specific threshold (percent identity: 75%; query coverage: 50%) against 18 919 full plasmid sequences (Fig. 4).

The highest proportion (described as frequency of the particular category divided by the number of all coding sequences identified within the studied sample) of classified proteins (8.6% for S9 and 4.4% for S11) was assigned to the category L “replication, recombination, and repair.” Other classified genes with high frequencies were related to the COG categories R “general function prediction only” (5.1% for S9 and 4.9% for S11), K “transcription” (3.8% for S9 and 2.2% for S11) and M “cell envelope biogenesis, outer membrane” (3.8% for S9 and 3.0% for S11). Our results revealed that the majority of genes of the Siberian metaplasmidomes shared similarities with genes present on plasmids originating from *Proteobacteria*. Single genes also shared similarities with genes of plasmids belonging to the phyla *Actinobacteria*, *Cyanobacteria*, *Deinococcus–Thermus*, *Firmicutes*, *Acinetobacteria*, and *Bacteroidetes*. A high proportion of plasmid-encoded genes in S9 (63.5%) and S11 (78.4%) could not be assigned to any described COG functional category.

### Plasmid-carried stress-tolerance genes

**Fig. 5:**
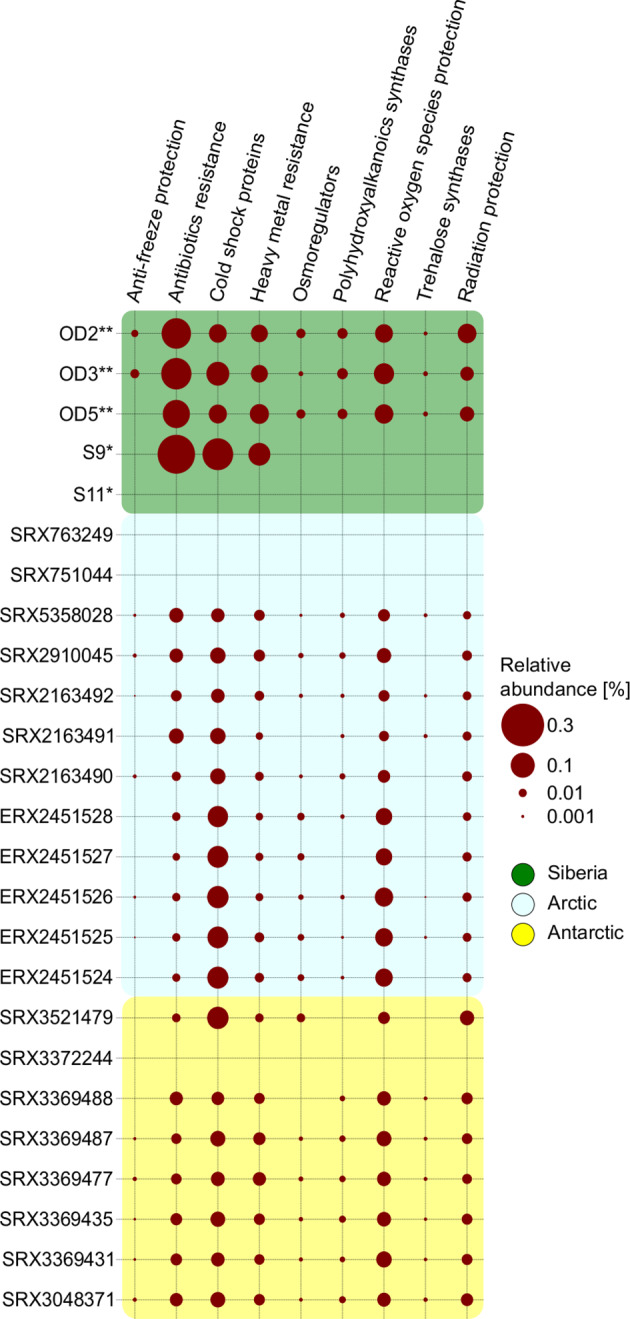
Stress-response-related gene distribution in enrichments and metagenomic datasets (threshold ident: 50.0%; qcov: 75.0%). The bubble size denotes the relative abundance of genes related to stress response found in metaplasmidomes from Siberia (green), Arctic plasmids from metagenomes (light blue), and plasmids from Antarctic metagenomes (light yellow). The size of the bubbles defines the relative abundance of stress-related genes observed among plasmidomes from Siberia and those retrieved from the Arctic and Antarctic metagenomic datasets. *corresponds to datasets obtained from enrichment cultures; **corresponds to Samoylov Island metagenomes.

The proportion of proteins assigned to particular groups of stress-related genes is depicted in Fig. 5. A high relative abundance (normalized by the number of all predicted proteins) of stress-response genes related to antibiotic resistance (0.3%; *H-NS*, *mcr-9*, and *yojI*), cold shock protection (0.2%; *cspB* and *cspL*), and heavy-metal resistance (0.1%; *czcA*) was found in the S9 Siberian metaplasmidome. Within S11 metaplasmidome, no stress-related gene was found.

The profile of stress-response genes was also analyzed in plasmid contigs extracted from Arctic and Antarctic metagenomes previously retrieved from the database. Besides genes related to antibiotic resistance, cold shock protection, and heavy-metal resistance, other genes encoding antifreeze proteins, cold shock proteins, reactive oxygen species and radiation protectants, osmoregulators, and polyhydroxyalkanoic and trehalose synthases were found. However, those genes contribute at most 0.2% to the metaplasmidome proteins per site (Fig. 5). In case of three Siberian metagenomes (OD2, OD3, and OD5) from Samoylov Island, the profiles of stress-response genes associated with plasmids were similar in terms of the presence and contribution of genes from a particular category (Supplementary Figure SI). The overall contribution of these genes did not exceed 0.4% and, interestingly, the most abundant were potential antibiotic resistance genes (OD2—0.2% of all genes identified in the metagenome dataset; OD3—0.2%; OD5—0.1%). The majority of predicted antibiotic resistance genes showed similarity to multidrug resistance pumps, such as *macB*, *msbA*, *mdtC*, *oleC*, *muxB*, and *triC*. In addition, potential aminoglycosides (*AAC(3)-Ic*, *AAC(6’)-Isa*, *AAC(6’)-Ib-SK*, *AAC(3)-Ib*), tetracycline (*tetA(48)*, *tetA(60) tet(30)*, *tetS*), and sulfonamide resistance gene (*sul4*) plus two hypothetical beta-lactamases (*bla*_JOHN-1_, *bla*_TLA-1_) were found. We also detected a large proportion of gene-encoding proteins potentially involved in cold shock protection, heavy-metal resistance, reactive oxygen species scavenging, and UV radiation protection. Amongst these stress-response genes, the following dominants were identified: *cspA* (3.5% of all stress-related genes identified from the Samoylov Island metagenomes), *infA* (2.3%), *arsA* (1.7%), *actP* (1.3%)*, sufS* (2.1%), *lipA* (2.1%), and *rlmN* (2.7%).

## Discussion

In this study, we isolated and analyzed metaplasmidomes of Siberian low-centered polygon tundra by applying a combination of enrichment culturing and massive plasmid isolation with high-throughput sequencing (Illumina). To our knowledge, this represents the first attempt to isolate an environmental pool of plasmids from Arctic soil characterized by extremely harsh environmental conditions.

The enrichment strategy described in this study aimed to obtain an adequate plasmid biomass for high-throughput sequencing even at the cost of general diversity loss of cultivated bacteria in relation to the raw sample. R2A nutrient solution was chosen as the growth medium for plasmid enrichment based on our preliminary analyses as it has previously reflected the broadest bacterial diversity of various soil microbiomes, including polar microbiomes [31, 51, 52]. A loss of bacterial diversity during cultivation was also demonstrated in other studies [51, 53–55]. The R2A enrichment of the soil was found to apply to *Proteobacteria*, *Bacteriodetes*, *Firmicutes*, and *Actinobacteria*, which is consistent with other studies [51, 56, 57].

During the enrichment, taxonomic composition significantly changed. This is a bias of the methodology, which promotes taxa (e.g., *Enterobacteriaceae*, *Moraxellaceae*) that are faster growing. It is also worth mentioning that a significant limitation of the applied methodology is that for a plethora of bacteria, the most suitable cultivation conditions (including medium composition) are still unknown. Comparing the taxonomic composition of initial samples with the enrichments, it was noted that some taxa were outnumbered. For two taxa (i.e., *Enterobacteriaceae* and *Moraxellaceae*), this significant increase in abundance may be explained by the fact that R2A medium is highly suitable for these taxonomic groups, and thus it may favor their growth. As for the *Firmicutes*, these are spore-forming bacteria, which may be problematic during environmental DNA isolation. It has already been noted that *Firmicutes* are known to be a dominant bacterial group in various soil environments, including extreme habitats [58, 59]. However, high-throughput metagenomic techniques may significantly underestimate their relative abundance since DNA isolation from spores is challenging [60]. We speculate that the application of an enrichment enabled spore germination, and thus, after this step, *Firmicutes* emerged in a large quantity.

In this study, it was indicated that the effectiveness of enrichment with R2A medium may be correlated with carbon, nitrogen, hydrogen, and sulfur content in the original soil sample. The S9 sample had higher content of these elements and the number of ASVs retrieved after enrichment was of 37.0% higher comparing with S11 sample, while in the original soil samples, 23.0% more ASVs was identified in S11 sample. This may be a consequence of “incongruity” between demands of bacteria inhabiting S11 (and potentially other environments with low C, N, H, S content) and R2A medium composition, which possibly results in the strong depletion of diversity and the change of dominant groups in such a sample after enrichment. However, this needs further investigations.

After an enrichment, the number of ASVs decreased more than ten times, while the structure of the bacterial community changed significantly. Therefore, metaplasmidomes obtained after enrichments do not fully correspond to the plasmidomes of the original soil samples. This is an important limitation of the presented methodology. Analyzing the potential hosts of plasmid contigs from Siberian metaplasmidomes, it was proposed that they belonged to *Moraxellaceae*, *Pseudomonadaceae*, *Enterobacteriaceae*, *Pectobacteriaceae, Burkholderiaceae*, and *Firmicutes*, which correlates with the taxonomic composition of the enrichments. However, a plethora of plasmid contigs were not assigned to any known taxa, which suggested that their hosts may still be unknown or there are too few sequence/genome records in the NCBI database for a given taxon.

It is also important to mention that a significant difference in the number of plasmids in two analyzed samples may result from the fact that, after enrichment, the richer sample (namely S9) was dominated by representatives of the family *Moraxellaceae* (24.9%). This family includes *Acinetobacter* and *Psychrobacter*, which are widely found in organic matter-rich environments, including Arctic and Antarctic soils, and majority of strains belonging to these genera contain multiple plasmids [61–63].

For an effective sequencing of plasmids from environmental samples, an efficient DNA isolation procedure needs to be applied. In this study, plasmid DNA isolation was performed by alkaline lysis using Plasmid Midi Kit (Qiagen). This was possible as sufficient biomass of bacterial cells was obtained during the enrichment. It is important to mention that direct isolation of plasmid DNA from raw Siberian soil samples was unsuccessful. A benefit of the applied methodology was also the fact that alkaline lysis protected the plasmid DNA from fragmentation and efficiently eliminated chromosomal DNA, which resulted in a higher proportion of plasmid contigs (in relation to chromosomal ones) and a higher contig length as compared with plasmid contigs retrieved from available metagenomes. Increased contig size, in turn, enabled analyzing the broader genomic context (e.g., co-localization) of identified genes, which is essential during the analysis of plasmids [64].

Within the identified plasmid contigs of the Siberian metaplasmidomes, few stress-response genes were identified. This may point out the role of plasmids in adaptation to challenging environmental conditions (such as low temperature). However, these genes are of a rather low relative abundance. Identification of the antibiotic resistance genes within plasmids of tundra soil-related bacteria is of high interest. We identified the presence of the *mcr-9* gene, which may confer resistance to colistin, recognized as a drug of last resort, and is critically important for the treatment of serious infections in an era of antibiotic crisis [65]. The presence of the antibiotic resistance genes at the S9 site may be correlated with the higher contribution of representatives of the *Enterobacteriaceae* family in this sample. This family of bacteria contains many pathogens frequently carrying antibiotic resistance genes. Following this idea, one may conclude that this is a consequence of zoonotic transmission, as bacteria belonging to this family are commonly found in gut microbiota of, for example, mammals or wild birds [66, 67]. Another possibility is that the presence of antibiotic resistance genes is a consequence of the close proximity of the Lena River, which is possibly a vector for transmission of various anthropogenic pollutants, including communal wastes and pathogenic bacteria, from remote urban areas of Russia [68] or airborne transmission, as it was proposed for bacteria worldwide [69, 70]. Finally, it is also possible that these genes are naturally present within Siberian bacteria as a response to naturally produced antibiotic-like compounds. Antibiotic-resistant genes have now been reported in various soil samples from Arctic and Antarctic regions, including millions-year-old permafrost, i.e., these genes originate from the pre-antibiotic era in medicine [24, 62, 71, 72]. Detection of antibiotic resistance genes in bacterial species recovered from permafrost indicates that antibiotic resistance predates humanity and these are natural resistance mechanisms to antimicrobial agents produced and used by microorganisms as weapons in inter-microbial competition within the biosphere [72].

In conclusion, we demonstrated that appropriate enrichment can reveal the potential of plasmids within the microbial community of active-layer tundra to serve as a model in studying the ecological role of plasmids in polar environments. The application of enrichment resulted in higher average contig lengths and a significantly higher recovery of plasmid sequences from assemblies as compared with metagenome-retrieved plasmids.

## Supplementary information

Figure SI
