# Supplementary material for: Metaplasmidome-encoded functions of Siberian low-centered polygonal tundra soils
Source: ISME J. 2021 May 19;15(11):3258–70. doi: 10.1038/s41396-021-01003-y (PMC8528913; doi:10.1038/s41396-021-01003-y)
Supplement: Supplementary file 1 — Figure SI [file 41396_2021_1003_MOESM1_ESM.pdf]

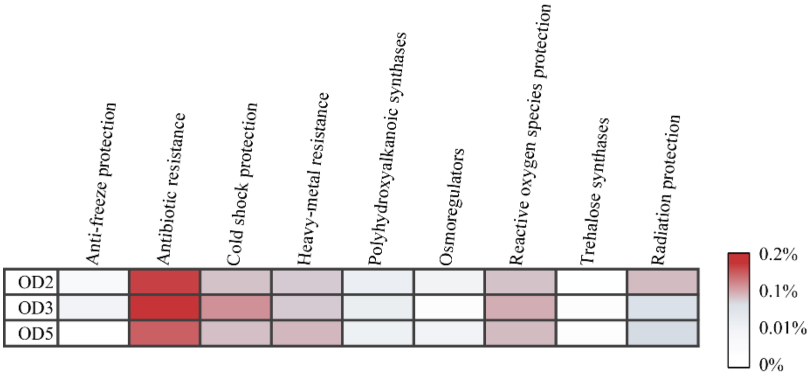

Figure SI. Stress response-related gene distribution in Siberian metagenome (from Samoylov Island) datasets (threshold pident: 50.0 %; qcov: 75.0%).
